# Congenital eyelid ptosis, decreased glomerular filtration, and orthostatic hypotension: Questions

**DOI:** 10.1007/s00467-016-3494-2

**Published:** 2016-11-17

**Authors:** Tessa Wassenberg, Michèl Willemsen, Henry Dijkman, Jaap Deinum, Leo Monnens

**Affiliations:** 1 Department of Neurology and Donders Institute for Brain, Cognition and Behaviour, Radboud University Medical Center, PO Box 9101, 6500 HB Nijmegen, The Netherlands; 2 Department of Pathology, Radboud University Medical Center, Nijmegen, The Netherlands; 3 Department of Internal Medicine, Radboud University Medical Center, Nijmegen, The Netherlands; 4 Department of Physiology, Radboud University Medical Center, Nijmegen, The Netherlands

**Keywords:** Orthostatic hypotension, Dopamine beta hydroxylase deficiency, Proximal tubule

## Case presentation

**Fig. 1:**
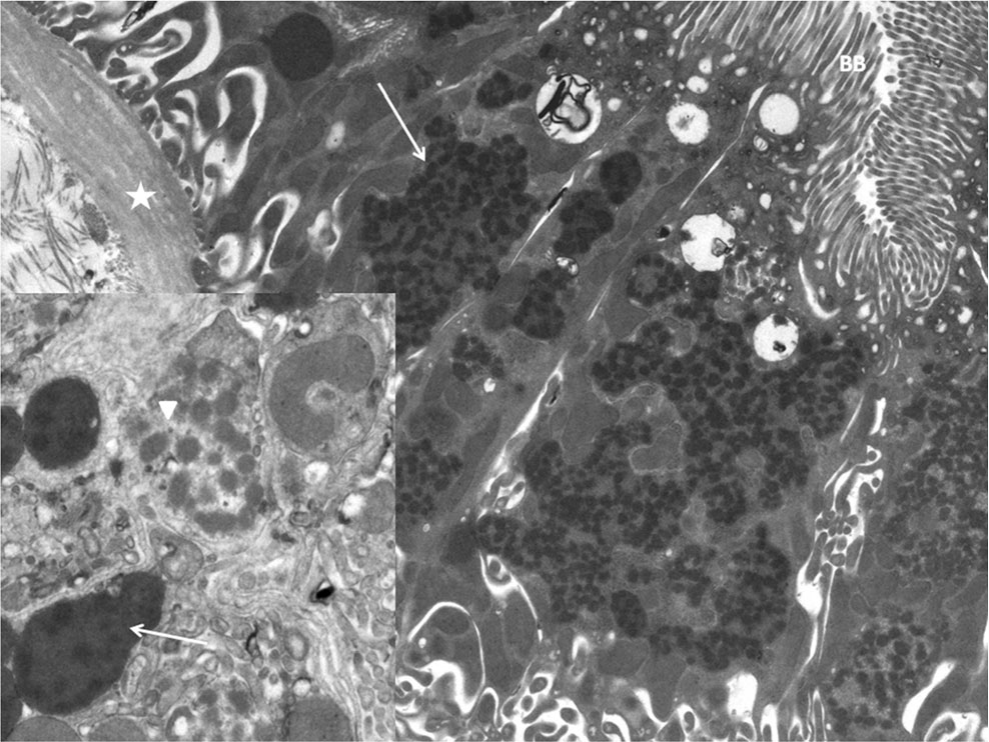
Electron microscopic image of kidney biopsy showing notable abnormal mitochondria. *Asterisk* Basal membrane, *BB* brush border proximal tubule, *arrow* fused mitochrondria. *Inset*: *Arrowhead* Swollen mitochondria exhibit electron-lucent areas, *arrow* fused mitochondria present amorphous matricial densities

An 18-year-old girl was referred to our department of pediatric nephrology due to elevated serum creatinine level. She was born premature and dysmature (gestational age 31 weeks + 5 days, birth weight 1210 g) to consanguineous parents. She had a good start but a very low blood glucose level directly after birth (0.2 mmol/L); this was corrected with an intravenous glucose infusion, and there was no further hypoglycemia in the neonatal period. She had mild bilateral eyelid ptosis from birth. Psychomotor development was normal. During childhood, she had a history of recurrent airway infections and bronchitis, mild failure to thrive, and mild anemia for which she visited a general pediatrician regularly. At age 4 years, routine serum creatinine measurements were 58 μmol/L, with an estimated glomerular filtration rate (eGFR) of 60 ml/min/1.73 m^2^. The lower than expected eGFR escaped the attention of the local pediatrician, and it was only at age 15 years, during routine testing, that a mildly elevated serum creatinine was noted for the first time (106 μmol/L, eGFR 53 ml/min/1.73 m^2^). At age 18 years, the serum creatinine level had increased to 139 μmol/L (eGFR 48 ml/min/1.73 m^2^), and she was referred to our university hospital for further examination. At that time, she reported exercise intolerance and lightheadedness on rising in the morning. Physical examination showed a height of 1.56 m (−2 standard deviations [SD]), a weight of 60 kg (+1SD), and a supine blood pressure of 106/55 mmHg; results were normal for heart, lungs, and abdomen examination, and, with the exception of the eyelid ptosis, for the neurological examination. Her symptoms and signs, especially the exercise intolerance and eyelid ptosis, raised the suspicion of a mitochondrial disorder. Laboratory investigations showed normal values for serum lactate and electrolytes (Na^+^ 140 mmol/L, K^+^ 4.4 mmol/L, Ca^2+^ 2.2 mmol/L, Mg^2+^ 0.75 mmol/L, P^3−^ 1.33 mmol/L). There was no proteinuria nor hematuria. Kidney size and structure were normal on the renal ultrasound images. In order to obtain a diagnosis, we performed a renal biopsy. Light microscopic examination revealed a few vascular sclerosed glomeruli with only discrete interstitial fibrosis. Electron microscopic examination revealed notable abnormal, fused mitochondria in the proximal tubules but not in the distal tubules (Fig. 1). This prompted the performance of a muscle biopsy for further evaluation of a mitochondrial defect. However, muscle histology was normal, and extensive biochemical testing of the respiratory chain also did not show any evidence of a mitochondrial disorder [1]. Mutation analysis of mitochondrial DNA was performed in leucocytes and muscle tissue, but did not show any abnormalities. At that time, current techniques, such as whole exome sequencing, were not yet available, and so the patient was managed further by our department without a classifying diagnosis. At age 19 years, she fainted while her height was being measured during a routine visit to the outpatient clinic. She reported that she was increasingly suffering from dizziness, especially in the morning. Orthostatic blood pressure measurements were performed, showing severe orthostatic hypotension with preserved heart rate increase. Her blood pressure decreased from supine values of 116/76 mmHg (heart rate 54 bpm) to 56/30 mmHg (heart rate 84 bpm) after standing for 3 min. Upon asking, she reported that sweating was normal.

## Questions

1. What diagnosis can be suspected and which tests can be used to confirm this?
2. What could be the explanation of the mitochondrial abnormalities in the proximal tubule (Fig. 1), and the decreased glomerular filtration rate in this patient?
3. What treatment is available?
